# Antibiotic burden of school children from Tibetan, Hui, and Han groups in the Qinghai–Tibetan Plateau

**DOI:** 10.1371/journal.pone.0229205

**Published:** 2020-02-24

**Authors:** Yushan Huang, Zuhong Zhang, Tianchun Hou, Jingfang Shi, Wenjie Huang, Zhao Bai, Danfeng Long, Xiaodan Huang, Shijuan Yan

**Affiliations:** 1 School of Public Health, Lanzhou University, Lanzhou, China; 2 Agro-biological Gene Research Center, Guangdong Academy of Agricultural Sciences, Tianhe District, Guangzhou, China; Addis Ababa University School of Public Health, ETHIOPIA

## Abstract

**Background:**

Given their geographical proximity but differences in cultural and religious dietary customs, we hypothesize that children from the three main ethnic populations (Han, Hui, and Tibetan) residing in the Qinghai–Tibetan Plateau region differs in their non-iatrogenic antibiotic loads.

**Methods:**

To determine the antibiotic burden of the school children unrelated to medical treatment, we quantified the antibiotic residues in morning urine samples from 92 Han, 72 Tibetan, and 85 Muslim Hui primary school children aged 8 to 12 years using high-performance liquid chromatography-tandem mass spectrometry, and performed correlation analysis between these data and concurrent dietary nutrition assessments.

**Results:**

Sixteen of the 18 targeted antibiotics (4 macrolides, 3 β-lactams, 2 tetracyclines, 4 quinolones, 3 sulfonamides, and 2 aminoanols) were identified in the urine samples with an overall detection frequency of 58.63%. The detection frequency of the six antibiotic classes ranged from 1.61% to 32.53% with ofloxacin showing the single highest frequency (18.47%). Paired comparison analysis revealed significant differences in antibiotic distribution frequency among groups, with Tibetans having higher enrofloxacin (*P* = 0.015) and oxytetracycline (*P* = 0.021) than Han children. Norfloxacin (a human/veterinary antibiotic) was significantly higher in the Hui children than in the Han children (*P* = 0.024). Dietary nutrient intake assessments were comparable among participants, showing adequate levels of essential vitamins and minerals across all three ethnic groups. However, significant differences in specific foods were observed among groups, notably in lower fat consumption in the Hui group.

**Conclusions:**

The introduction and accumulation of antibiotic residues in school children through non-iatrogenic routes (food or environmental sources) poses a serious potential health risk and merits closer scrutiny to determine the sources. While the exact sources of misused or overused antibiotics remains unclear, further study can potentially correlate ethnicity-specific dietary practices with the sources of contamination.

## Introduction

Antibiotics are widely used in clinical and veterinary practices, animal husbandry, and aquaculture. Inevitably, these antibiotics are transmitted to the environment and passed to the human body through many possible routes. Veterinary antibiotics may enter the human body via contaminated water and food, resulting in the accumulation of non-iatrogenic antibiotics in the body [1]. Additionally, iatrogenic misuse of antibiotics, such as preventive usage, improper choices of antibiotics, and incorrect administration or dosage, can also lead to adverse conditions among patients [2, 3]. The increasing use of antibiotics for therapeutic as well as non-therapeutic purposes in the animal food industry is also an serious issue globally due to the dissemination of antibiotic resistance genes and the leaching of antibiotics into the environment [4]. For example, Wang *et al*. (2015) found location-specific differences in antibiotic exposure due to (mis)management of clinical antibiotic use, accumulation of residues in food, and agricultural and industrial dispersal of residues to the environment [5].

According to unofficial statistics, China is among the top nations for antibiotic production and use, potentially resulting in overuse and bacterial resistance [6]. Children are reportedly more susceptible to the effects of environment and food-borne antibiotic residues than adults, which even in a low doses may lead to adverse effects [7] such as allergic reaction, organ damage, and intestinal dysbacteriosis. Furthermore, antibiotic misuse promotes the emergence of resistant pathogens that can weaken immunity, reduce host resistance to self-limited diseases, and potentially cause super infections [4, 7]. Although humans may experience exposure to low doses of antibiotic residues in food or water, even short-term exposure is accompanied by health risks [8, 9]. Previous studies have shown that even short-term antibiotic exposure can create a strong selective pressure for resistant intestinal flora, the effects of which can endure for several years following treatment [10, 11]. Several cohort studies in infants found an association between cumulative exposure to antibiotics in early life, especially broad-spectrum, and obesity in later years [12, 13]. The sources of exposure may be complex in nature.

Preliminary evidence has indicated that antibiotic exposure may be a pressing issue even among relatively isolated populations such as those living in the Qinghai–Tibetan Plateau region. The Qinghai–Tibetan Plateau in western China is the world’s largest and the highest plateau. A wide diversity of ethnic groups reside on the Qinghai–Tibetan Plateau, despite its lagging economic development and severe climate. Some populations in this rural region are nomadic and subsist by grazing yak and sheep. The three largest ethnic groups are Han, Hui, and Tibetan, which vary in their lifestyle and living environment, social structure, religious and cultural practices, and local diet. Several previous studies have evaluated the health among human population on the plateau, especially children [14–16]. The traditional diets of each group also differ, notably in the consumption of pork and the sources of meat and dairy.

In addition, different ethnic groups show range of attitudes toward seeking medical treatment for children. For example, Tibetan children may generally receive traditional treatments unless they are seriously ill [17]. Although school children may be particularly sensitive to improper antibiotic exposure, few studies have examined the role of antibiotics on the health of school children, and the differences in exposure between ethnic groups in the Qinghai–Tibetan Plateau [14–17].

This study investigated the antibiotic burden of school children from the Han, Hui, and Tibetan groups in the Qinghai–Tibetan Plateau and examined potential differences in this burden between groups. The goal of this work is to provide a quantitative description of the antibiotic load among participants and to possibly identify the sources of antibiotic residues that are potentially related to dietary and cultural practices specific to respective ethnic groups. The results of this study can provide valuable resource for identifying health issues related to antibiotic usage and bacterial resistance in humans. This work also provides a basis for understanding potential risks that are unique to specific ethnic groups to improve treatment strategies and overall food safety.

## Materials and methods

All procedures performed were approved by the Medical Ethics Committee of school of public health in Lanzhou University. Participants received detailed information about the study and informed signature consent was obtained from by a parent/legal guardian and the administrator/ teacher of the local primary school.

### Study site and population

The Gannan Tibetan autonomous prefecture is located at the eastern edge of the Qinghai–Tibetan Plateau and is populated with 24 different ethnic groups [18]. Xiahe County (lon 102°31'25", lat 35°12'9") in the Gannan Tibetan Autonomous Prefecture was selected as the study site.

Prior to starting the full study, we gathered preliminary data through a questionnaire and biological samples from 40 school children who all attended the Railway NO.1 school in Lanzhou at the eastern edge of the Qinghai–Tibetan Plateau. These initial screens indicated that the lowest rate of occurrence of residual antibiotics in the urine of these children was approximately 59% in the Han group, which was then used as the estimated value for the rate of antibiotic residues, the allowable error was controlled within 50% (the actual value was 11%). Thus, the formula for calculation of sample size is as follows:

$$\text{N}=\left(\frac{u_{\alpha }\times \pi \times \left(1-\pi \right)}{\delta^{2}}\right)=\left(\frac{1.96^{2}\times 0.59\times \left(1-0.59\right)}{0.11^{2}}\right)=77$$

We thus determined the total sample size needs to be at least n = N × 3 = 231.

Approximately 300 out of 1200 candidate third to fifth graders, aged 8 to 12 years, from the Labrang Primary School were selected as the study participants using a multi-stage stratified cluster sampling method. The participants volunteered with the consent of their guardians. Basic information on the participants (such as gender and age) and their family background (*e*.*g*., education levels and occupation of their guardians) were obtained from the school's admission records and through a questionnaire survey (S1 File). The weight and height of participants were measured on the site. In addition, information about the frequency and quantity of antibiotic administration for participants was noted in the questionnaire. Participants with missing demographic information on age, gender, or ethnic group as well as those who received antibiotics treatment within the past 3 months were excluded from the study. Finally, 249 school children (92 Han, 72 Tibetan, and 85 Muslim Hui) were enrolled as participants in this study.

### Urine sample collection

Sample collection was conducted in October of 2017. Morning urine samples were collected from the 249 participants individually under the instructions of the investigators and stored in 50 mL sample bottles with 50 μL 0.05% sodiumazide. Samples were temporarily stored in boxes containing dry ice, and immediately transported to the laboratory for storage at -80°C until further analysis.

### Selection of antibiotics

In order to identify possible non-iatrogenic sources of antibiotics for school children, we first investigated which main types of antibiotics were used in local farms. A total of 18 antibiotics were selected and measured in this study including four macrolides (azithromycin, clarithromycin, erythromycin, and roxithromycin), three β-lactams (penicillin, amoxicillin, and tylosin), two tetracyclines (oxytetracycline and tetracycline), four quinolones (ofloxacin, ciprofloxacin, enrofloxacin, and norfloxacin), three sulfonamides (sulfadimidine, methoxybenzidine, and sulfadiazine), and two aminoanols (chloroamphenicol and olaquindox). Among these antibiotics, six were used only for treatment of humans (azithromycin, clarithromycin, roxithromycin, norfloxacin, ofloxacin, chloroamphenicol), three for veterinary treatments only (enrofloxacin, tylosin, olaquindox), and the rest for both human and veterinary treatments.

### Antibiotic quantification by HPLC-MS/MS

Prior to initiating the experiment, we established a detailed method of detection for sample determination [19]. A 0.5 mL aliquot of each urine sample was extracted with 3 μL formic acid and 1 mL 100% methanol, with shaking on a thermoshaker for 30 min (1000 rpm, 20°C). Then, the mixture was centrifuged and the upper layer was transferred to a roQ QuEChERS d-SPE tube (200 mg) (Phenomenex, Torrance, CA). After vortexing for 5 min and centrifuging for 10 min (14,000 rpm, 4°C), after which 200 μL aliquots of the final extraction solution were transferred to vials for HPLC-MS/MS analysis. From each extracted sample, 5 μL were injected into a reversed-phase Gemini NX-C18 column (00B-4453-B0, 3 μm, 50 × 2 mm) (Phenomenex, Torrance, CA). The antibiotic compounds were separated by reversed-phase ultra-fast LC (Shimadzu, Kyoto) and detected using an electrospray ion source tandem triple quadrupole API4000 MS analyzer (ABSciex, Foster City, CA). The quantification of multiple samples was conducted following previously established parameters for LC and MS described by Huang *et al*. (2019) [19]. The Analyst 1.5.2 software (ABSciex, Singapore) was used to control the instrument and for data acquisition and processing of MS peak spectra.

### Statistical analysis

The dietary survey was based on previously published food frequency questionnaires for dietary assessment [20, 21]. The subject height was measured by mechanical altimeter, and subjects were weighed with an RGZ-120 electronic scale (Suhong Co. Ltd, China) with kilogram readings measured to one decimal. EpiData 3.1 software was used for double entry, and SPSS20.0 was used for statistical analysis. All data were tested for normal distribution, and appropriate corresponding statistical methods were selected according to the distribution.

Differences in the frequency of urinary antibiotics detection among the three ethnic groups were tested by Chi-square test, and all subsequent pairwise comparisons among subgroups were further conducted by extracting individual cases. The test levels were: *a* = 0.01 and 0.05; β = 0.1. Furthermore, in order to reduce the probability of making type I errors in the pairwise comparisons, *a* was corrected as 0.05/3. In addition, considering that urinary antibiotic residues may originate in specific foods, we developed logistic regression models based on the food intake and urinary antibiotic values in the school-age children in order to explore the potential relationship between dietary intake and antibiotic residues (S4 Table).

### Investigation of dietary nutrition and physical development

Dietary nutritional status of the patients was investigated using the pre-prepared questionnaire. The dietary questionnaire (S1 File) was divided into three parts; one for the participants and two for their respective guardians, and all questionnaires were filled out with the help of our research members after a unified training. The semi-quantitative food frequency method [20, 21] was adopted to estimate the types or varieties of food consumed over the past 6 months. The 72 hours food recall [22] was used to collect the intake levels of all food varieties and nutritional quality at school and at home 3 days before the sampling of urine. The dietary intakes of the 249 school children from the six months preceding this study were then evaluated in accordance with the *Dietary Guidelines for Chines*e *Residents* [23]. Nutrient analysis was carried out for each subject using CDGSS3.0 nutrition software. All the participants were assessed for anthropometric measurement according to the Chinese national standard GB/T31178—2014 “*Comprehensive evaluation of the development level of children and adolescents*” procedure and WHO Child Growth standards “*Length/height-for-age(HAZ)*, *weight-for-age(WAZ)*, *weight-for-height or body mass index-for-age(WHZ or BAZ) methods and development”* [24].

## Results

### General information about subjects

A total of 249 school children aged 8–12 years old were enrolled in this study, with an average age of 10.00 ± 1.23 years. There were 72 Tibetan children (37 boys and 35 girls), 85 Hui children (48 boys and 37 girls), and 92 Han children (42 boys and 50 girls).

### General characteristics of urinary antibiotics

**Fig 1:**
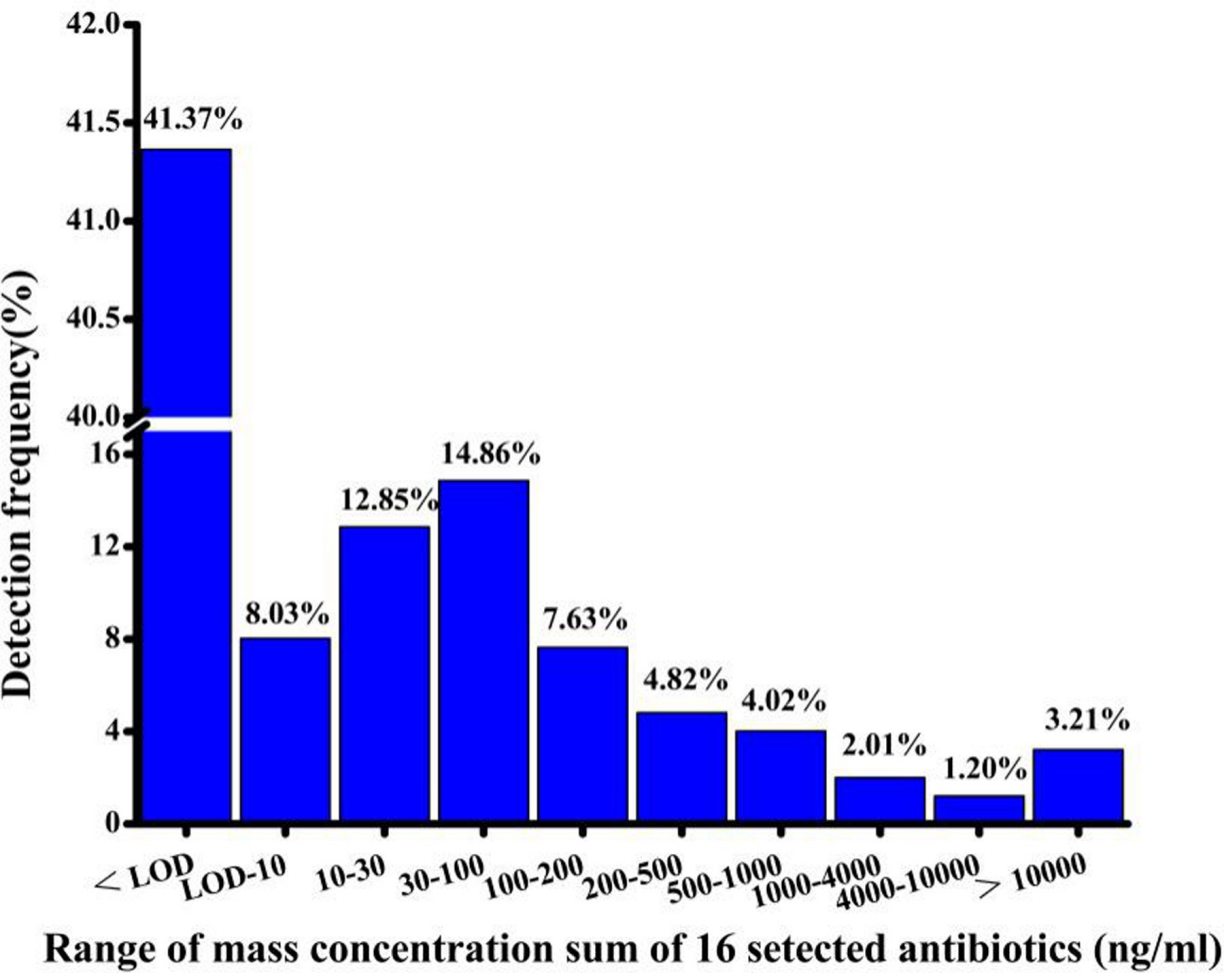
Distribution of mass concentration detection frequencies for 16 selected antibiotics (LOD: Limit of detection).

Tylosin and olaquindox (typically used for veterinary applications) were not detected among the 18 antibiotics, whereas the detection frequency of the remaining 16 antibiotics ranged from 0.4% for sulfadiazine to 18.47% for ofloxacin (Table 1). The overall detection frequency of the 16 antibiotics was 58.63%, and residues of β-lactams, macrolides, quinolones, sulfonamides, tetracyclines, and aminoanol ranged from 1.61% to 32.53%. Azithromycin, ciprofloxacin, ofloxacin, tetracycline, and oxytetracycline were detected in more than 10% of the urine samples. Eight of the 16 antibiotics were found in particularly high concentrations of greater than 1500 ng/mL. Ofloxacin had the highest detection frequency of 18.47% among the six human antibiotics, and ciprofloxacin had the highest detection frequency of 16.47% among the nine human/veterinary antibiotics. Meanwhile, enrofloxacin was the only animal antibiotic purely used for animals, which had a detection rate of 3.61% (Table 1). The detection frequencies of all of the antibiotics were within the concentration ranges of 0.85 (minimum)–10.0, 10.0–30.0, 30.0–100.0, and 10,000–711,011.1 ng/mL (maximum), and at these concentrations were detected at a rate of 8.03%, 12.85%, 14.86%, and 3.21%, respectively (Fig 1). Additionally, we found two types of antibiotics combined in 40 subjects (13.6%), three types in 20 subjects, four types in 10 subjects, and five types in 2 subjects. One child was exposed to 7 types of antibiotics from macrolides, quinolones, sulfonamides, and tetracyclines.

**Table 1:** Descriptive analysis of selected antibiotics in all subjects (n = 249).

Antibiotic(ng/ml)	Usage	n(%)	Percentile					Max
			50th	75th	90th	95th	99th	
**β-lactams (β-LAC)**	—	20 (8.03)	—	—	—	16.124	204348.95	711000.00
**Amoxicillin (AMO)**	Human/veterinary	14 (7.23)	—	—	—	15.70	204301.50	711000.00
**Penicillin (PEN)**	Human/veterinary	4 (1.61)	—	—	—	—	0.8505	94.90
**Macrolides (MA)**	—	40 (16.06)	—	—	62.50	847.50	21326.55	120660.00
**Azithromycin (AZI)**	Human	29 (11.65)	—	—	7.020	62.10	6790.00	10900.00
**Erythromycin (ERY)**	Human/veterinary	13 (5.22)	—	—	—	78.05	6340.00	11900.00
**Clarithromycin (CLA)**	Human	19 (7.63)	—	—	—	183.00	6095.00	10200.00
**Roxithromycin (ROX)**	Human	5 (2.01)	—	—	—	—	666.500	18300.00
**Quinolones (QUI)**	—	81 (32.53)	—	13.15	60.70	116.00	1905.55	711000.00
**Enrofloxacin (ENR)**	Veterinary	9 (3.61)	—	—	—	—	25.25	70.40
**Ciprofloxacin (CIP)**	Human/veterinary	41 (16.47)	—	—	11.90	23.80	310.50	494.00
**Norfloxacin (NOR)**	Human/veterinary	6 (2.41)	—	—	—	—	435.50	3200.00
**Ofloxacin (OFL)**	Human/veterinary	46 (18.47)	—	—	35.10	54.70	135.50	711000.00
**Sulfonamides (SAS)**	—	9 (3.61)	—	—	—	—	52.20	290.00
**Sulfamethazine (SUL-M)**	Human/veterinary	5 (2.01)	—	—	—	—	18.20	113.00
**Sulfadiazine (SUI-D)**	Human/veterinary	1 (0.40)	—	—	—	—	—	16.20
**Trimethoprim (TRI)**	Human/veterinary	5 (2.01)	—	—	—	—	47.15	177.00
**Tetracyclines (TCs)**	—	60 (24.10)	—	—	89.30	176.44	1456.00	408000.00
**Tetracycline (TET)**	Human/veterinary	25 (10.04)	—	—	10.70	105.00	1448.50	408000.00
**Oxytetracycline (TER)**	Human/veterinary	40 (16.06)	—	—	12.00	40.30	279.00	562.00
**Amphenicols (AMP)**	—	4 (1.61)	—	—	—	—	36.45000	603.000
**Chloroamphenicol (CHL)**	Human	4 (1.61)	—	—	—	—	36.45000	603.000
**All antibiotics (All-anti)**	—	146 (58.63)	10.1	83.450	483.0	1890.15	559528.1	711011.1

### Antibiotic residues in urine categorized by human use, veterinary use, or both human and veterinary use

**Fig 2:**
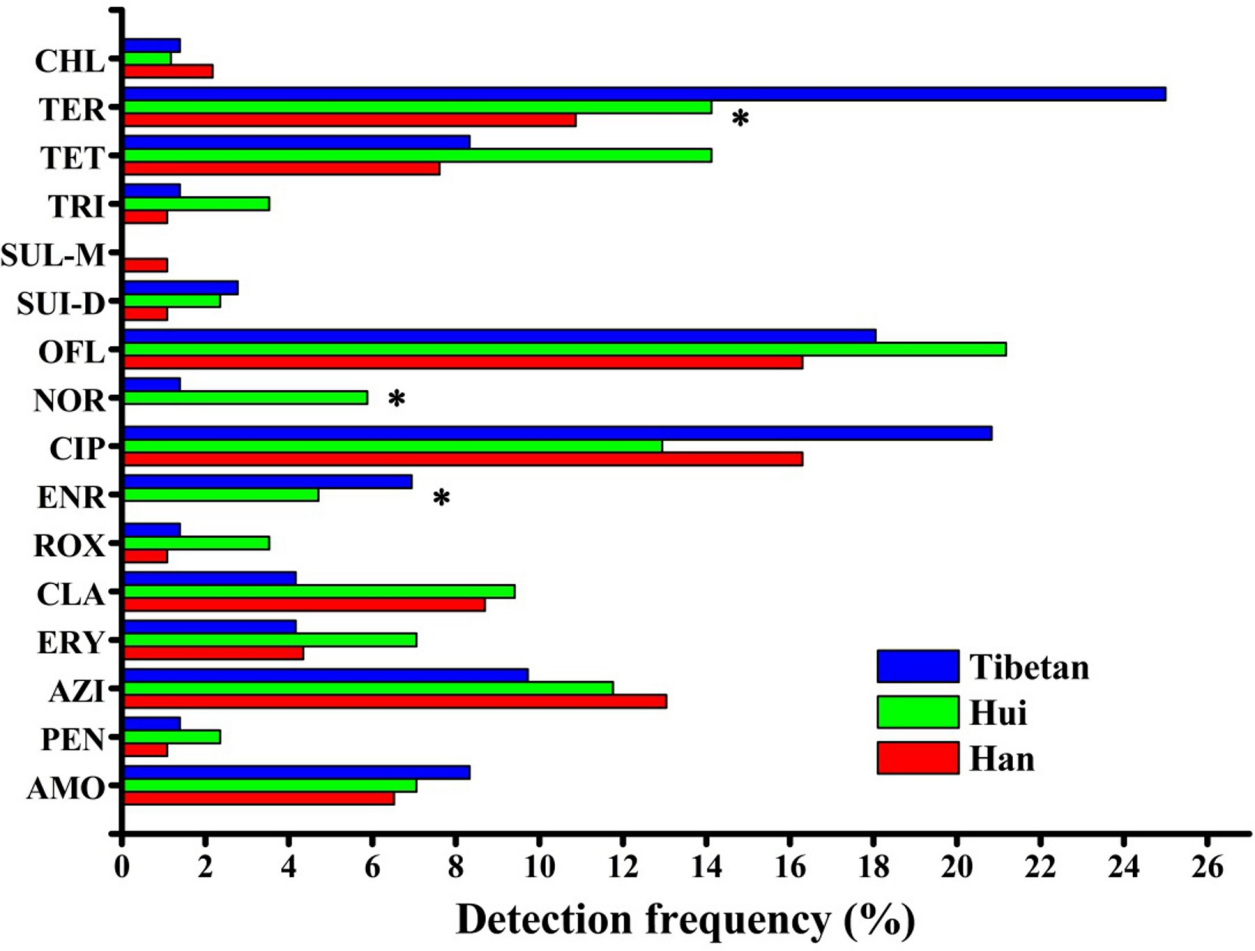
Detection frequency of antibiotics by antimicrobial mechanism in relation to ethnic categories (* indicates *P* < 0.05).

The detection frequencies of norfloxacin (*P* = 0.020), enrofloxacin (*P* = 0.021) and oxytetracycline (*P* = 0.042) were significantly different among the three ethnic groups. Paired comparison analysis showed that the distribution of norfloxacin was significantly higher in the Hui children than in the Han children (*P* = 0.024), although its distribution in the Hui and Han groups was not significantly different from that in the Tibetan children (Fig 2). The distributions of enfloxacin (*P* = 0.015) and oxytetracycline (*P* = 0.021) in the Tibetan children were higher than that in the Han children (Fig 2), while other pairwise comparisons of their distributions among ethnic groups showed no significant differences. The detection frequencies of human, veterinary, and human/veterinary antibiotics were 3.61%, 31.33%, and 44.18%, respectively (S1 Table).

### Antibiotics in urine categorized by antimicrobial mechanism

**Fig 3:**
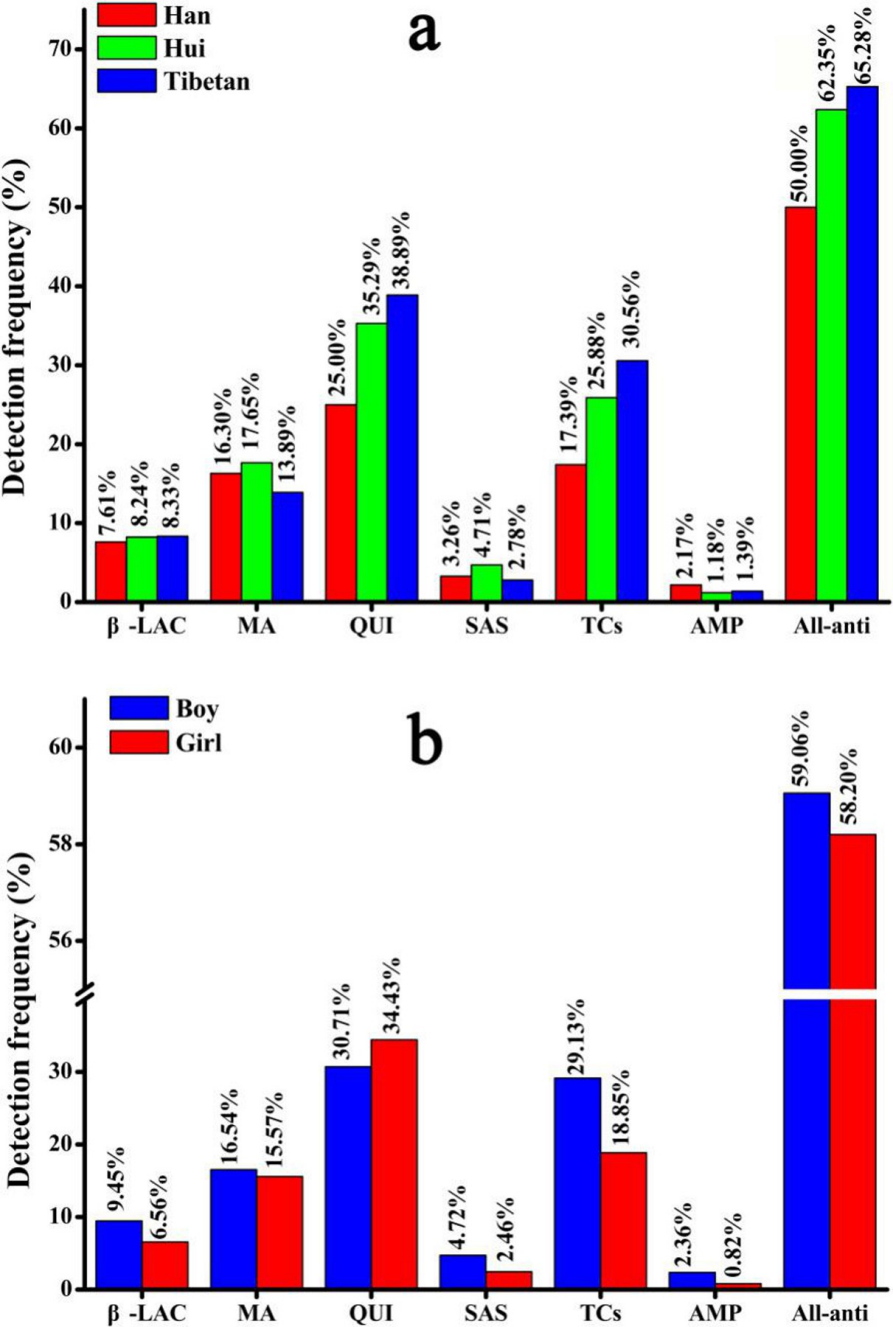
Detection frequency of antibiotic categories by antimicrobial mechanism in relation to (a) ethnic group and (b) sex. (* indicates *P* < 0.05).

The detection frequencies in urine samples for each of the six categories of antibiotics were analyzed to identify potential relationships between residue accumulation and ethnicity or gender. Results showed no significant differences in the urinary antibiotic detection frequencies for any of the six antibiotic categories among any of the three ethnic groups (Fig 3A), with the highest detection rates observed for quinolones and the lowest for amphenicol antibiotics. Similarly, analysis of gender groups revealed no significant differences in the detection frequencies of the six antibiotic categories between boys and girls (Fig 3B). However, the distribution of frequencies for all antibiotics among the three ethnics groups was significantly different (*P* < 0.05) (Fig 3A).

**Fig 4:**
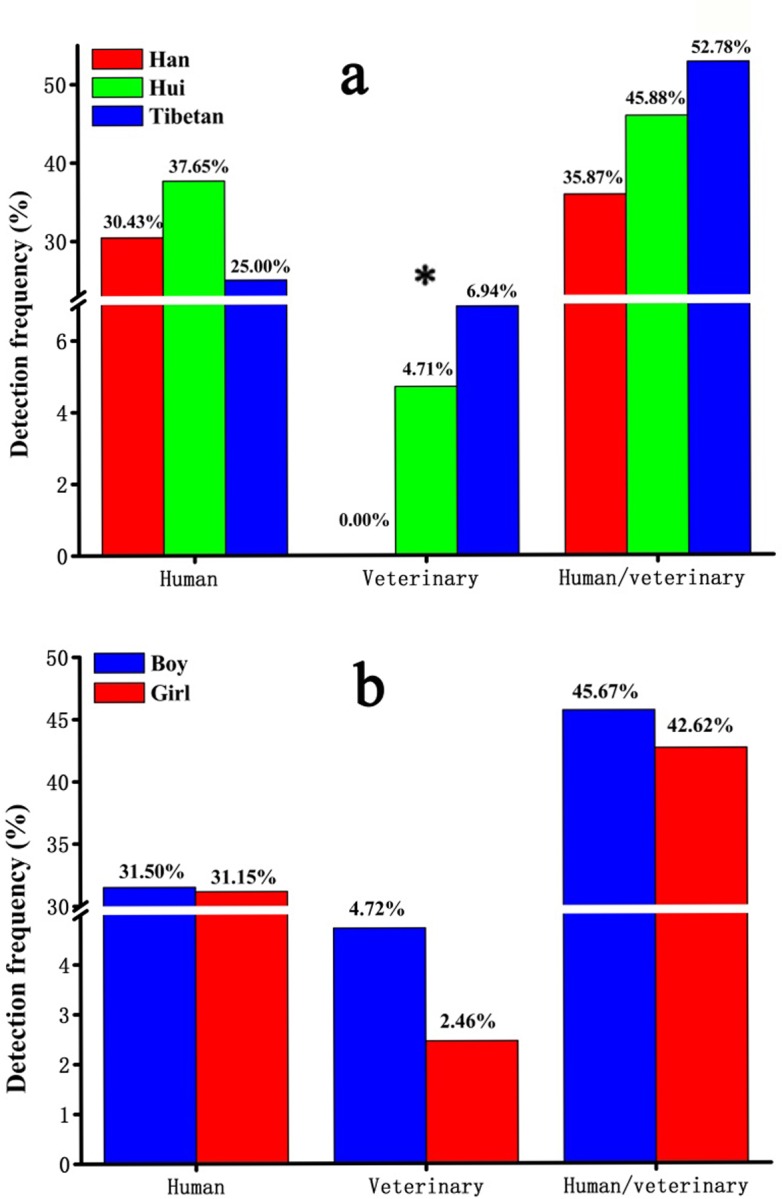
Detection frequency of antibiotics categorized by antibiotics categorized by human treatment, veterinary treatment, or usage for either in relation to (a) ethnic group and (b) sex (* indicates *P* < 0.05).

The detection frequencies (Fig 4) of antibiotics by designated usage (human, veterinary, or both) showed that antibiotics used for veterinary treatments were higher (*P =* 0.022) in urine samples from the Tibetan children (6.94%) than in those from the Han children (0.00%) (Fig 4A), whereas the detection frequency of these mixed use antibiotics among Hui children was 4.71%, with no significant differences between genders (Fig 4B).

### Evaluation of dietary nutrition and physical development

Based on data collected through the dietary questionnaire, the food types consumed by the school children were divided into nine categories and compared between ethnic groups (Table 2). These comparisons revealed that the Hui children had lower intake of vegetables and fish and prawn than the Han children (*P* < 0.01). The Tibetan children consumed greater amounts of milk and dairy products than the Hui children (*P* < 0.01). The Hui and the Tibetan children consumed lower amounts of beans than the Han children (0.01 < *P* < 0.05). Based on the differences in dietary intake and antibiotic residues among school-age children of the three ethnicities, we also produced logistic regression models that revealed Human antibiotics (*P* = 0.035), Quinolones (*P* = 0.046)and Tetracyclines (*P* = 0.035) residues in urine were statistically significant (S4 Table). Human antibiotic residues are related to dairy (β = -0.003, *P* = 0.002) and aquatic products (β = 0.008, *P* = 0.028). Dairy products and quinolone antibiotics (β = -0.002, *P* = 0.023), tetracycline antibiotics (β = -0.002, *P* = 0.031) residues have a close relationship (S4 Table).

**Table 2:** Average daily food intake among children from three ethnic groups. (Median (P_25_, P_75_)).

Food type	RNI (g)	Han (n = 92)	Hui (n = 85)	Tibetan (n = 72)
		Intake (g)	Intake (g)	Intake (g)
**Grains**	150–200	152.50 (85.75~260.00)	114.13 (76.00~268.56)	105.88 (72.13~223.00)
**Vegetables**	300	141.45 (57.53~251.44)^a^	93.45 (36.79~224.13)^b^	97.00 (57.36~179.89)^ab^
**Fruits**	150–200	102.31 (51.47~234.50)	95.25 (32.79~222.80)	91.22 (43.64~194.30)
**Meat and poultry**	40	24.51 (8.19~53.29)	14.38 (5.88~51.06)	26.97 (6.62~59.80)
**Fish and prawn**	40	2.78 (0.34~13.23)^a^	0.74 (0.00~4.48)^b^	1.78 (0.00~8.34)^ab^
**Milk and dairy products**	300	81.25 (34.00~206.00)^ab^	64.25 (22.38~127.94)^a^	144.25 (50.0~269.13)^b^
**Eggs**	25–40	7.57 (0.00~19.68)	9.86 (0.00~30.09)	8.81 (0.16~22.71)
**Beans**	105	15.00 (0.00~61.50)^a^	5.00 (0.00~34.00)^b^	8.50 (0.00~36.00)^b^
**Condiments and others**	-	91.39 (38.75~268.00)	57.38 (22.19~243.00)	77.75 (34.25~171.75)

^a.b^ superscript letters indicate a significant difference compared to other mean values within the same row (*P* < 0.05).

Due to the results of the data quality assessment template generate using the WHO Anthro Survey Analyser, we obtained the Z-score distribution of indicators and thus determined the underweight rate, emaciation rate, overweight rate, and growth retardation rate for all of the children in each of three ethnic groups (S2 Table). Statistical analysis and all pairwise comparisons showed no significant differences in physical development among the groups.

We used 24 nutrient indicators (S3 Table) in this study to dissect and compare the dietary nutritional intake of the children. Among the 24 indexes of nutrients, the intake of total fat significantly differed among the three ethnic groups (*P* < 0.01)with Tibetan and Han children exhibiting significantly higher fat intake than Hui children (Table 3). When the fat category was further divided into animal and vegetable fats, the Tibetan and Han children were similarly shown to have higher fat intake than the Hui children irrespective of plant or animal sources (*P* < 0.01) (Table 3). In addition, significantly different intake levels of vitamin E and iodine were found among the ethnic groups. Specifically, Han children consumed more vitamin E and iodine than the Hui children, who consumed significantly more carotene and iodine than the Tibetan children (S3 Table).

**Table 3:** Distribution table of fat daily intake for children from three ethnic groups. (M (P_25_, P_75_)).

Source	Han (n = 92)	Hui (n = 85)	Tibetan (n = 72)
	Intake (g)	Intake (g)	Intake (g)
**Total fat**	75.95 (46.87~103.42)^a^	47.86 (30.93~77.02)^b^	60.05 (40.30~103.52)^a^
**Animal fat**	21.86 (9.76~41.40)^a^	11.87 (6.88~25.76)^b^	20.52 (9.85~38.00)^a^
**Plant fat**	41.18 (26.42~67.41)	32.36 (19.36~57.99)	39.42 (22.38~58.11)

^a-b^ superscripts indicates a significant difference between mean values within a row (*P* < 0.05).

## Discussion

We screened for residues of 18 antibiotics from six categories based on their common usage in medical and veterinary treatments and livestock production. The antibiotics were detected in the morning urine samples of 249 (92 Han, 85 Hui, and 72 Tibetan) school children aged 8 to 12 years old from a primary school in the Xiahe County of the Gannan Tibetan autonomous prefecture, China. Except for tylosin and olaquindox, 16 antibiotics were detected in the urine samples from the study participants. Given that our study excluded children who were administered antibiotics within three months of sampling, these data strongly suggest that more than half of the children in the Autonomous Prefecture were exposed to residues through food or environmental sources resulting in a clear and distinct antibiotic body burden.

In general, clinical use, self-medication, and ingesting contaminated drinking water or food are the primary human exposure pathways to antibiotics [25–28]. The finding of antibiotics in food is far from new, and since the 1940s, antibiotics have been used extensively in animal husbandry and aquaculture [27]. In the USA, 70% out of a total of 16,200 tons of antibiotics have been used for livestock farming [29], resulting in high levels of antibiotic residues in meat, milk, and aquatic products [30]. Considering the distinct cultural and dietary practices of the three ethnic groups, we further examined the antibiotic load in relation to the dietary nutrition of the participants in order to determine whether not certain foods could be correlated with this phenomenon. Although in the present study, nutritional analysis (S3 Table) indicated comparable and sufficient dietary nutrition and essential vitamins and minerals among participants and across all three ethnic groups, we observed significant differences in the frequency of antibiotic distribution across ethnic groups and postulated that these differences were primarily related to dietary habits.

For example, the sources of dietary fat for Hui children, which was significantly lower than in Tibetan and Han children (*P* < 0.01), are mainly beef, mutton, and poultry; whereas the two other ethnic groups have more diverse sources of animal fats, including poultry, seafood, and pork. In the Qinghai–Tibetan Plateau, cattle and sheep are generally grazing ruminants, while poultry and pigs are kept in intensive production systems in which antibiotics are often used to prevent epidemics, promote growth, and improve feed efficiency. Considering this assumption, that more of their staple meats may have been raised in intensive feeding operations, we expected that the Tibetan children would carry a higher load of antibiotics in their body than the other ethnic groups. As expected, the concentration of veterinary antibiotics was significantly higher in the urine of the Tibetan children than in that of the Han children, while that of the Hui children was intermediate. This finding suggests that antibiotic exposure among the Tibetan children in this study is likely related to their high consumption of livestock meat and milk. To our knowledge, the majority of Tibetan children in this study were from families associated with yak and sheep farming, and consumed more meat and milk than Han and Hui children. In contrast, the Han consumed pork as a primary animal protein, significantly more than other groups, especially Hui, for whom pork is prohibited by religious tradition. According to the results of logistics regression models, milk and dairy products presented significantly association with antibiotic residues such as Quinolones and Tetracyclines, which also suggested possible antibiotic residue during dairy production and thus should be constantly concerned [31, 32].

We found a higher distribution of the veterinary antibiotic norfloxacin among Hui children (5.88%), which was significantly higher than in Han (0.00%) children and Tibetan children (1.39%). The Hui also had a higher consumption of poultry meat, whereas Han children consumed significantly greater amounts of vegetables, aquatic products, and beans than the Hui children. The use of ofloxacin and norfloxacin for food animal production was banned in China in 2015, but these antibiotics have been detected in recent studies. Wang *et al*. (2015) reported that ofloxacin was detected in more than 10% of 1046 school children, while norfloxacin was found in 3.5% [5], in agreement with our results showing a higher rate of ofloxacin detection than norfloxacin across groups. Given the potentially limited sources of halal poultry meat, and lower consumption of other meats compared to other groups the likelihood that the norfloxacin is present in the meat prior to consumption bears closer scrutiny, as has been reported in earlier studies on antibiotic contamination [28].

Antibiotic residues may come from environmental sources in addition to residues in food. Various antibiotics have been found in water and sediment substrates, with high concentrations of human antibiotics downstream of waste-water treatment plants, and veterinary antibiotics found in areas with significant agricultural activity [28]. Historically, norfloxacin, has been widely used in chicken farms [12]. Detection of the banned norfloxacin in this study suggests that awareness of the consequences of antibiotic misuse in remote regions may be absent, which is an issue requiring urgent public education and effective monitoring [33]. The β-lactams and macrolides are the two most widely administered classes of antibiotics in children, and they account for the majority of antibiotic use in this population [34, 35]. Except for some urgent cases, such as severe bacterial infections and when there are no better alternative antibiotics, quinolones are not used for children under 18 years of age because of possible cartilage and joint toxicity [36, 37].

Surprisingly, the detection rate of ofloxacin was generally higher than that of many other human/veterinary antibiotics among the study participants. Furthermore, in light of the side effects of enamel hypoplasia and irreversible staining of deciduous teeth, tetracyclines are not recommended for children under 8 years of age [37, 38]. Similarly, sulfonamides have been largely replaced by the safer β-lactams or macrolides in practice [34, 35]. However, these antibiotics are still used in animals for growth promotion, disease prevention, as feed additives, or for treatment of infections [6]. In addition, most of them are frequently detected in surface water or food in China [25, 26]. Based on the results of the dietary survey, we suspect that the source of tetracyclines, quinolones, and sulfonamides in the urine samples are not medical in nature, but instead from environmental contaminants or as residues in food.

Further evidence that the antibiotics observed in the study subjects did not originate in clinical treatments can be found in the inconsistent combinations of drugs found within individual samples or groups. That is, the number of combinations of mixed exposures to antibiotics and antibiotic categories found in this study considerably exceeded the number of clinically rational combined modes [39, 40]. Furthermore, the antibacterial effect of macrolides can potentially antagonize that of quinolones, and the antibacterial effect of sulfonamides may be independent of that of macrolides or quinolones in pharmacology [39]. This result suggests possible misuse or overuse of antibiotics clinically or in self-medication, and/or, based on similar findings in other studies, they come from environmental or food sources. In the present study, nutrition analysis (S3 Table) indicated that dietary nutrition among most participants was adequate and provided sufficient levels of essential vitamins and minerals across all three ethnic groups.

To the best of our knowledge, this study is the first to examine the antibiotics load and its relationship with diet in school children from different ethnic groups in the rural eastern edge of the Qinghai–Tibetan Plateau. Considering the widespread use of antibiotics in medical treatment, animal husbandry, and aquaculture, we selected 18 antibiotics as the targets of detection according to their increased probability of exposure among children [41–44]. We used UPLC-TOF-MS to determine the type and concentration of antibiotic residues in the urine samples due to its high sensitivity and reliability. Although the study was conducted at a specific geographical site of the Qinghai–Tibetan Plateau, results of this study can be broadly applied to other regions in China as well as other nations where antibiotic contamination poses a public health risk.

However, our evaluation of the antibiotic load in children was based on only 18 target antibiotics, and likely represents a conservative estimate for the wider population. It is altogether possible that considerably more varieties than we have tested in this study can be found in animal products and public water supplies. Given our unexpected finding of several agricultural antibiotics, the full scale of environmental antibiotic contamination thus warrants a more comprehensive assessment.

## Conclusion

Approximately 58.63% of the school children in the Gannan Tibetan autonomous prefecture at the eastern edge in the Qinghai–Tibetan Plateau had one or more probable non-iatrogenic antibiotic residues in their bodies. We found a clear trend in our samples of combined human and veterinary antibiotic residues that likely originated from food sources. In light of their role as a potential source of inadvertent antibiotic consumption, meat and dairy products will necessarily be tested as an important part of our ongoing research. However, long persistence after clinical treatment and environment contamination are other potential sources of the antibiotic residues observed in this study, and will be further scrutinized along with market samples of meat, seafood, and vegetables.

## Supporting information

S1 File (RAR)

S1 Table Descriptive analysis of antibiotic categories by usage in all subjects (n = 249). (DOCX)

S2 Table Evaluation results of growth and development levels of three ethnic children. (DOCX)

S3 Table Daily intake of dietary nutrients among children from three ethnic groups. (M (P_25_, P_75_)).
(DOCX)

S4 Table Results of *logistic regression* model of dietary intake and urine antibiotic detection in school-age children of three ethnics. (DOCX)
