# Supplementary material for: Antibiotic burden of school children from Tibetan, Hui, and Han groups in the Qinghai–Tibetan Plateau
Source: PLoS One. 2020 Feb 24;15(2):e0229205. doi: 10.1371/journal.pone.0229205 (PMC7039500; doi:10.1371/journal.pone.0229205)
Supplement: S1 Table — (DOCX) [file pone.0229205.s002.docx]

**Table S1. Descriptive analysis of antibiotic categories by usage in all subjects (n=249)**

| **Antibiotic (ng/ml)** | **N (%)** | **Percentile** | | | | | **Max** |
| --- | --- | --- | --- | --- | --- | --- | --- |
|  |  | **50th** | **75th** | **90th** | **95th** | **99th** |  |
| **Human** | 78 (31.33) | — | 21.60 | 123.00 | 902.00 | 19776.55 | 21100.0 |
| **Veterinary** | 9 (3.61) | — | — | — | — | 25.25 | 70.40 |
| **Human/veterinary** | 110 (44.18) | — | 22.85 | 174.23 | 505.35 | 408047.45 | 711000.0 |
