# Supplementary material for: Antibiotic burden of school children from Tibetan, Hui, and Han groups in the Qinghai–Tibetan Plateau
Source: PLoS One. 2020 Feb 24;15(2):e0229205. doi: 10.1371/journal.pone.0229205 (PMC7039500; doi:10.1371/journal.pone.0229205)
Supplement: S2 Table — (DOCX) [file pone.0229205.s003.docx]

**Table S2. Evaluation results of growth and development levels of three ethnic children.**

|  | **Han(n=92)** | **Hui(n=85)** | **Tibetan (n=72)** |
| --- | --- | --- | --- |
| **Underweight (%)** | 0 | 3.53 | 0 |
| **Growth retardation (%)** | 1.09 | 0 | 0 |
| **Emaciation (%)** | 7.61 | 9.76 | 4.17 |
| **Overweight (%)** | 2.17 | 0 | 2.78 |
