# Supplementary material for: Antibiotic burden of school children from Tibetan, Hui, and Han groups in the Qinghai–Tibetan Plateau
Source: PLoS One. 2020 Feb 24;15(2):e0229205. doi: 10.1371/journal.pone.0229205 (PMC7039500; doi:10.1371/journal.pone.0229205)
Supplement: S3 Table — (M (P25, P75)). (DOCX) [file pone.0229205.s004.docx]

**Table S3. Daily intake of dietary nutrients among children from three ethnic groups. (M (P_25_, P_75_))**

| **Nutrient** | **RNI** | **Han (n=92)** | **Hui (n=85)** | **Tibetan (n=72)** |
| --- | --- | --- | --- | --- |
|  |  | **Intake (g)** | **Intake (g)** | **Intake (g)** |
| Calorie (kcal)  Protein (g)  Fat (g)  Carbohydrate (g)  Dietary fiber (g)  Retinol equivalent (µg)  Thiamine (mg)  Riboflavin (mg)  Niacin (mg)  Vitamin C (mg)  Vitamin E (mg)  Kalium (mg)  Natrium (mg)  Calcium (mg)  Magnesium (mg)  Iron (mg)  Manganese (mg)  Zinc (mg)  Copper (mg)  Phosphonium (mg)  Selenium (mg)  Iodine (mg) | 1450–2050  40–60  /  120–150  /  500  1.0  1.0  10–14  65–90  9–13  1500–1900  1200–1400  1000–1200  220–300  13–18  3.0–4.0  7.0–10.0  0.5–0.7  470–640  40–55  90–110 | 2089.34(1376.93~2809.01)  71.40(45.32~103.42)  75.95(46.87~103.42)^a^  267.37(159.70~407.40)  14.00(8.40~24.20)  562.20(259.70~928.60)  0.80(0.50~1.20)  1.10(0.70~1.70)  17.70(10.20~24.90)  93.00(55.50~172.30)  35.00(21.30~58.40)^a^  2647.30(1567.80~4156.10)  1807.70(1284.40~3201.90)  610.50(410.00~1061.90)  399.50(237.40~570.30)  24.40(16.50~44.10)  6.00(3.00~15.60)  10.60(7.30~17.20)  2.40(1.50~3.70)  989.30(632.00~1511.10)  46.70(26.30~72.10)  161.20(53.30~455.00)^a^ | 1909.42(926.31~2976.64)  52.00(29.82~103.79)  47.90(30.93~77.02)^b^  240.89(160.47~430.46)  12.75(5.85~23.00)  317.40(180.08~800.78)  0.80(0.40~1.28)  0.90(0.50~1.68)  13.10(7.73~27.43)  78.10(40.15~154.18)  25.85(14.23~43.20)^b^  2348.95(1319.68~4570.05)  1923.60(1241.08~2598.55)  556.10(307.00~1024.38)  411.65(170.55~603.78)  23.45(12.03~44.88)  4.65(2.30~11.00)  10.10(4.93~17.93)  1.85(0.80~3.60)  908.60(436.38~1452.13)  38.55(23.78~70.83)  58.30(39.95~91.43)^b^ | 1869.90(1185.77~2721.78)  61.37(33.95~90.20)  228.32(145.08~428.55)^a^  60.05(40.23~103.52)  10.25(7.20~16.80)  351.25(214.48~663.25)  0.80(0.40~1.10)  1.05(0.58~1.70)  14.20(8.20~23.85)  82.20(40.95~135.38)  27.20(17.15~49.80)^a^  2383.25(1318.48~3412.53)  1907.20(1335.35~2566.93)  632.00(357.23~976.80)  356.30(205.88~490.43)  20.30(13.15~33.60) 4.10(2.78~7.65)  10.25(5.50~14.43)  1.90(1.28~3.55)  1024.30(584.25~1438.35)  48.85(29.30~88.53)  59.15(45.03~93.10)^b^ |

RNI (recommended nutrient intake), a nutrient intake level that can meet the needs of the vast majority of individuals (97%–98%) in a given gender, age, and physiological status group.[23] ^a-b^ indicates values within a row with a different superscript are significantly different (*P*<0.05).
