# Supplementary material for: Antibiotic burden of school children from Tibetan, Hui, and Han groups in the Qinghai–Tibetan Plateau
Source: PLoS One. 2020 Feb 24;15(2):e0229205. doi: 10.1371/journal.pone.0229205 (PMC7039500; doi:10.1371/journal.pone.0229205)
Supplement: S4 Table — (DOCX) [file pone.0229205.s005.docx]

**Table S4. Results of *Logistic Regression* model of dietary intake and urine antibiotic detection in school-age children of three ethnics.**

***Logistic Regression* Block 1: Method = Enter**

| **Variables** | **Omnibus Tests of Model Coefficients** | | | **Hosmer and Lemeshow Test** | | | **Classification Table^a^** | |
| --- | --- | --- | --- | --- | --- | --- | --- | --- |
|  | **Model** | | |  |  |  | **Predicted** | |
|  | **Chi-square** | **df** | **Sig.** | **Chi-square** | **df** | **Sig.** | **Overall Percentage** | **Percentage Correct** |
| Human/veterinary antibiotics | 8.535 | 10 | 0.577 | 6.376 | 8 | 0.605 | 56.9% | |
| Human antibiotics | 19.427 | 10 | 0.035* | 6.513 | 8 | 0.590 | 71.8% | |
| Veterinary antibiotics | 14.518 | 10 | 0.151 | 9.198 | 8 | 0.326 | 96.4% | |
| β-lactams | 3.625 | 10 | 0.963 | 8.633 | 8 | 0.374 | 91.5% | |
| Macrolides antibiotics | 7.370 | 10 | 0.690 | 5.949 | 8 | 0.653 | 84.7% | |
| Quinolones | 12.622 | 10 | 0.046* | 13.300 | 8 | 0.102 | 69.4% | |
| Sulfonamides | 8.858 | 10 | 0.546 | 7.897 | 8 | 0.444 | 96.8% | |
| Tetracyclines | 11.098 | 10 | 0.035* | 10.785 | 8 | 0.214 | 77.0% | |
| Amphenicols | 12.737 | 10 | 0.239 | 0.926 | 8 | 0.999 | 98.8% | |

^a^.The cut value is 0.500.

*.*P* < 0.05 indicates that in the fitted model, the OR value of at least one of the included variables has statistical significance, that is, the overall significance of the model.

**Variables in the Equation**

Dependent variable: Human antibiotics

| **Step 1^a^** | **β** | **S.E.** | **Wald** | **df** | **Sig.** | **Exp(β)** | **95% C.I. for EXP(β)** | |
| --- | --- | --- | --- | --- | --- | --- | --- | --- |
|  |  |  |  |  |  |  | **Lower** | **Upper** |
| Race 1 |  |  | 0.117 | 2 | 0.943 |  |  |  |
| Race 2 | 0.093 | 0.339 | 0.076 | 1 | 0.783 | 1.098 | 0.565 | 2.133 |
| Race 3 | -0.021 | 0.366 | 0.003 | 1 | 0.955 | 0.979 | 0.478 | 2.008 |
| Grains | 0.000 | 0.001 | 0.061 | 1 | 0.805 | 1.000 | 0.998 | 1.002 |
| Milk and dairy products | -0.003 | 0.001 | 9.580 | 1 | 0.002* | 0.997 | 0.995 | 0.999 |
| Eggs | 0.007 | 0.006 | 1.747 | 1 | 0.186 | 1.007 | 0.996 | 1.018 |
| Meat and poultry | 0.001 | 0.001 | 0.378 | 1 | 0.539 | 1.001 | 0.999 | 1.003 |
| Fish and prawn | 0.008 | 0.004 | 4.818 | 1 | 0.028* | 1.008 | 1.001 | 1.015 |
| Beans | 0.000 | 0.002 | 0.012 | 1 | 0.911 | 1.000 | 0.996 | 1.004 |
| Vegetables | -0.001 | 0.001 | 0.945 | 1 | 0.331 | 0.999 | 0.997 | 1.001 |
| Fruits | 0.000 | 0.000 | 0.643 | 1 | 0.423 | 1.000 | 0.999 | 1.001 |
| Constant | -0.605 | 0.303 | 3.993 | 1 | 0.046 | 0.546 |  |  |

^a^.Variable(s) entered on step1: Race1,2,3, grains, milk and dairy products, eggs, meat and poultry, fish and prawn, beans, vegetables, fruits.

**Variables in the Equation**

Dependent variable: Quinolones

| **Step 1^a^** | **β** | **S.E.** | **Wald** | **df** | **Sig.** | **Exp(β)** | **95% C.I. for EXP(β)** | |
| --- | --- | --- | --- | --- | --- | --- | --- | --- |
|  |  |  |  |  |  |  | **Lower** | **Upper** |
| Race 1 |  |  | 0.225 | 2 | 0.894 |  |  |  |
| Race 2 | -0.099 | 0.335 | 0.087 | 1 | 0.769 | 0.906 | 0.470 | 1.747 |
| Race 3 | 0.070 | 0.353 | 0.040 | 1 | 0.842 | 1.073 | 0.537 | 2.143 |
| Grains | 0.001 | 0.001 | 1.571 | 1 | 0.210 | 1.001 | 0.999 | 1.003 |
| Milk and dairy products | -0.002 | 0.001 | 5.197 | 1 | 0.023* | 0.998 | 0.996 | 1.000 |
| Eggs | 0.004 | 0.005 | 0.664 | 1 | 0.415 | 1.004 | 0.994 | 1.015 |
| Meat and poultry | 0.000 | 0.001 | 0.380 | 1 | 0.537 | 1.000 | 0.999 | 1.002 |
| Fish and prawn | 0.005 | 0.003 | 2.148 | 1 | 0.143 | 1.005 | 0.998 | 1.011 |
| Beans | 0.000 | 0.002 | 0.034 | 1 | 0.854 | 1.000 | 0.996 | 1.003 |
| Vegetables | -0.001 | 0.001 | 1.133 | 1 | 0.287 | 0.999 | 0.997 | 1.001 |
| Fruits | 0.001 | 0.001 | 0.731 | 1 | 0.393 | 1.001 | 0.999 | 1.002 |
| Constant | -0.713 | 0.296 | 5.805 | 1 | 0.016 | 0.490 |  |  |

*.*P*<0.05 indicates statistical significance.

**Variables in the Equation**

Dependent variable: Tetracyclines

| **Step 1^a^** | **β** | **S.E.** | **Wald** | **df** | **Sig.** | **Exp(β)** | **95% C.I. for EXP(β)** | |
| --- | --- | --- | --- | --- | --- | --- | --- | --- |
|  |  |  |  |  |  |  | **Lower** | **Upper** |
| Race 1 |  |  | 1.243 | 2 | 0.537 |  |  |  |
| Race 2 | -0.396 | 0.367 | 1.165 | 1 | 0.280 | 0.673 | 0.328 | 1.382 |
| Race 3 | -0.276 | 0.387 | 0.509 | 1 | 0.476 | 0.759 | 0.356 | 1.619 |
| Grains | 0.000 | 0.001 | 0.218 | 1 | 0.641 | 1.000 | 0.999 | 1.002 |
| Milk and dairy products | -0.002 | 0.001 | 4.637 | 1 | 0.031* | 0.998 | 0.996 | 1.000 |
| Eggs | 0.008 | 0.006 | 1.780 | 1 | 0.182 | 1.008 | 0.996 | 1.019 |
| Meat and poultry | 0.000 | 0.001 | 0.001 | 1 | 0.976 | 1.000 | 0.998 | 1.002 |
| Fish and prawn | 0.006 | 0.003 | 3.587 | 1 | 0.058 | 1.006 | 1.000 | 1.013 |
| Beans | 0.003 | 0.003 | 1.267 | 1 | 0.260 | 0.997 | 0.992 | 1.002 |
| Vegetables | 0.000 | 0.001 | 0.084 | 1 | 0.772 | 1.000 | 0.999 | 1.001 |
| Fruits | 0.000 | 0.000 | 0.918 | 1 | 0.338 | 1.000 | 1.000 | 1.001 |
| Constant | -0.845 | 0.309 | 7.471 | 1 | 0.006 | 0.430 |  |  |

*.*P*<0.05 indicates statistical significance.
